# Avian phenotypic convergence is subject to low genetic constraints based on genomic evidence

**DOI:** 10.1186/s12862-020-01711-7

**Published:** 2020-11-07

**Authors:** Yu-Chi Chen, Hao-Chih Kuo, Wen-Sui Lo, Chih-Ming Hung

**Affiliations:** 1 Biodiversity Research Center, Academia Sinica, Taipei, Taiwan; 2 Department of Evolutionary Biology, Max Planck Institute for Developmental Biology, Tübingen, Germany

**Keywords:** Convergent evolution, Genomic comparison, Genetic constraint, Nocturnal birds, Foot-propelled diving birds, Raptors

## Abstract

**Background:**

Phenotypic convergence between distinct species provides an opportunity to examine the predictability of genetic evolution. Unrelated species sharing genetic underpinnings for phenotypic convergence suggests strong genetic constraints, and thus high predictability of evolution. However, there is no clear big picture of the genomic constraints on convergent evolution. Genome-based phylogenies have confirmed many cases of phenotypic convergence in birds, making them a good system for examining genetic constraints in phenotypic convergence. In this study, we used hierarchical genomic approaches to estimate genetic constraints in three convergent avian traits: nocturnality, raptorial behavior and foot-propelled diving.

**Results:**

Phylogeny-based hypothesis tests and positive selection tests were applied to compare 16 avian genomes, representing 14 orders, and identify genes with strong convergence signals. We found 43 adaptively convergent genes (ACGs) associated with the three phenotypic convergence cases and assessed genetic constraints in all three cases, from (amino acid) site mutations to genetic pathways. We found that the avian orders shared few site mutations in the ACGs that contributed to the convergent phenotypes, and that these ACGs were not enriched in any genetic pathways. In addition, different pairs of orders with convergent foot-propelled diving or raptorial behaviors shared few ACGs. We also found that closely related orders that shared foot-propelled diving behavior did not share more ACGs than did distinct orders, suggesting that convergence among these orders could not be explained by their initial genomic backgrounds.

**Conclusions:**

Our analyses of three avian convergence events suggest low constraints for phenotypic convergence across multiple genetic levels, implying that genetic evolution is unpredictable at the phylogenetic level of avian order. Ours is one of first studies to apply hierarchical genomic examination to multiple avian convergent cases to assess the genetic constraints in life history trait evolution.

## Background

Stephen Jay Gould argued that, if the tape of life could be replayed, the outcome would be different every time [1]. This suggests that evolution is unrepeatable, but cases of phenotypic convergence—distinct lineages independently reaching a similar phenotype [2]—have long fascinated biologists because they seem to contradict Gould’s hypothesis. While the genetic mechanism underlying phenotypic convergence is largely unclear, it is critical to understanding how evolution operates. For example, investigating how species converge genetically may help us evaluate whether evolution is subject to strong constraints and is thus predictable [3–5], i.e., whether evolution is governed solely by probabilities or confined to a limited number of genetic variables rendering predictable phenotypic outcomes [6, 7].

The debate over the molecular underpinnings of phenotypic convergence has lasted for decades [4]. Stayton [8] used a simulation to show that simple traits can converge by chance. However, actual cases of convergence often involve traits that are coded by complex genetic pathways, making it unclear whether most examples of convergence occur by chance. On the other hand, one genetic mutation may impact multiple traits that have antagonistic effects on the organism’s fitness (i.e., pleiotropic effect), and thus the number of effective mutations could be restrained [9]. Therefore, authors argue that different species may find the same genetic solutions in response to similar environmental pressures, causing adaptive convergence [10, 11]. If this is the case, then another question is whether genetic convergence tends to occur at the level of (nucleotide or amino acid) site mutations, individual genes or genetic pathways.

The evolution of convergence in complex traits cannot be fully understood using conventional candidate gene approaches, but instead requires genome-wide analyses [12]. Liu et al. [13] found that bats and toothed whales share 14 derived amino acids in one motor protein, *Prestin*, which is important for sensing ultrasound and is assumed to be critical for the organisms’ convergent echolocation trait. However, Parker et al. [12] used genome sequence data to show that signatures of convergence between bats and toothed whales are detected in up to 200 genes, many of which are linked to hearing, deafness or vision. Therefore, genome-wide analyses provide a new avenue to examine the molecular mechanisms of phenotypic convergence [14–16] and identify novel genes associated with complex traits, even for non-model species.

The whole-genome sequences of many taxa, including almost every avian order, were recently published [17, 18], making it possible to extensively test for genome-wide signatures of convergent evolution. Avian phylogenies based on the above data [17] or genome-wide data from most avian families [19] confirm that there are many cases of phenotypic convergence in birds. Thus, birds provide a good system to examine genomic constraints in phenotypic convergence. In this study, we explore the genomic bases of avian convergence on three traits—nocturnality, raptorial behavior and foot-propelled diving. These three convergent traits are diverse and occur in various parts of the avian phylogeny, and we chose them to generate a broad view of the genetic mechanisms underlying phenotypic convergence among avian orders.

Avian lineages with each of the three convergent traits are widely distributed across all continents except the Antarctic, where only a few species occur [20]. Thus, the convergence of these traits occurs in lineages that are generally sympatric. Early molecular studies based on DNA–DNA hybridization suggested that owls were sister to nightjars and their allies, all of which are active at night [21, 22]. More recent studies have shown that the nocturnality is convergent in owls (234 owl species constituting the order Strigiformes [20]) and nightjars (98 nightjar species and 34 species of their nocturnal allies constituting five families in the order Caprimulgiformes [20]) because they belong to distinct lineages and thus evolved the trait independently [23–25]. Owls are more closely related to Coraciimorphae birds such as woodpeckers than to nightjars; nightjars are more closely related to hummingbirds, both of which are Caprimulgimorphae birds, than to owls [17]. Thus, owls and nightjars represent a typical case of convergent evolution [23]. Until around two decades ago, diurnal raptors—birds of prey that generally have hooked beaks and taloned feet and are active in the daytime—were considered to be monophyletic [26–28]; however, recent research suggests that diurnal raptors should be divided into two non-sister groups: falcons (66 falcon species constituting the order Falconiformes [20]) and other diurnal raptors (252 eagle, hawk or kite species constituting the order Accipitriformes and seven New World vulture species constituting its sister order, Cathartiformes [20]) [25]. Falcons are more closely related to parrots and passerines, all of which belong to Australaves, than to other diurnal raptors, which belong to Afroaves [17]. Thus, the evidence suggests that raptorial traits shared between falcons and other diurnal raptors have evolved independently.

Grebes (22 species constituting the order Podicipediformes [20]), loons (5 species constituting the order Gaviiformes [20]) and cormorants (40 cormorant species and their foot-propelled diving allies, including four anhinga species, constituting two families in the order Suliformes [20]), also phylogenetically distinct, independently acquired similar foot-propelled diving traits [17, 23]. Loons and grebes were once treated as each other’s closest relatives [29], partly because they both use feet instead of wings to propel through the water and have rearward positioned legs; however, three decades ago, genetic evidence revealed that the two groups are distinct [21]. Cormorants are another avian lineage characterized by foot-propelled diving behavior, but they are also not sister to grebes or loons [19].

Interestingly, loons are phylogenetically closer to cormorants [17], but more similar to grebes in morphology and behavior [30] because the two groups faced similar selective forces. In general, phylogenetically more closely related species share more similar genomic background and ancestral alleles [31], and are thus predicted to achieve convergent traits via more similar genetic bases [32]. Therefore, the foot-propelled diving birds provide a good system to test whether selective forces can surpass phylogenetic closeness and cause adaptive genes to reoccur. Note that, because no study has performed ancestral state reconstruction for nocturnality, raptorial behavior or foot-propelled diving, we cannot rule out the possibility that these traits were present in the common ancestors of the studied lineages and subsequently lost in other lineages. However, we assume that the focal traits in the studied lineages converged because it is the most parsimonious explanation.

**Fig. 1:**
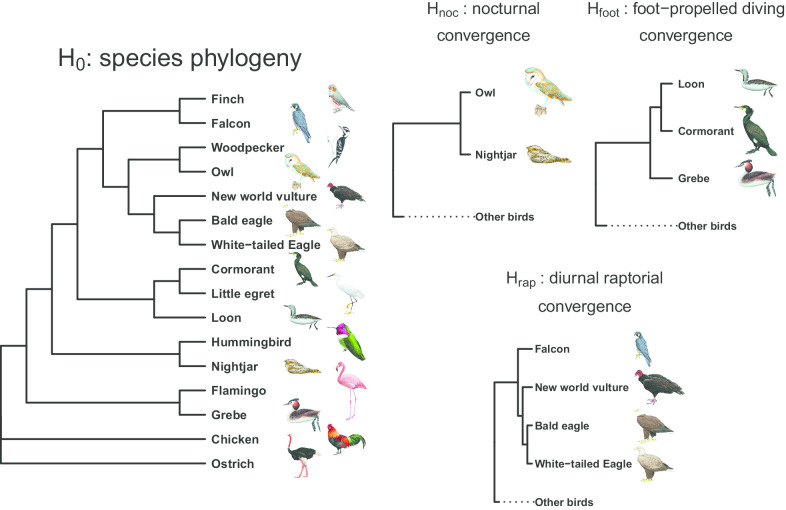
Hypotheses on genomic sequence convergence. The species phylogeny of the focal species is based on Jarvis et al. [17] and was used as the null hypothesis. The three alternative convergence hypotheses, H_noc_, H_foot_ and H_rap_, forced nocturnal, foot-propelled diving and raptorial birds to form monophyletic clades, respectively (All bird illustrations were reproduced with permission from Lynx Edicions)

In this study, we conducted hierarchical genomic analyses, including phylogeny-based hypothesis tests, positive selection tests and enrichment tests, on the three convergence cases—nocturnality, raptorial behavior and foot-propelled diving—across avian orders (Fig. 1). We aimed to answer two specific questions: (1) Do the three convergent traits show high or low genetic constraints at the different hierarchical levels from site mutations to genetic pathways? (2) Is it phylogenetically or ecologically closer pairs that share more genes associated with the foot-propelled diving convergence? The results help us evaluate the predictability of evolution, especially evolution of the molecular underpinnings of complex traits. In addition, we demonstrate that analyses framed by convergent genome comparison can also help identify candidate genes for phenotypic traits in non-model species.

## Results

### Identification of adaptively convergent genes

We obtained the genome sequences of 16 study species (Table 1), representing 14 avian orders, from an avian genomic project database [17, 33, 34] and chose 4278 orthologous genes containing at least partial sequences for all study species for downstream analyses. We developed a two-step approach to identify “adaptively convergent genes (ACGs)” that might contribute to the convergent traits among the focal taxa. The approach was modified from Parker et al.’s [12] approach, which was criticized by two studies [35, 36]; we used these critiques to improve the approach by establishing reasonable null models for assessing the statistical significance of sequence convergence signals and adding a second step to detect positive selection signals in genes with convergent sequences to enable the identification of ACGs (see “Methods” for details).

**Table 1:** The 16 avian genomes used in this study

Common name	Scientific name	Trait of interest	References
Anna’s hummingbird	*Calypte anna*	Related to nightjars^a^	Zhang et al. [33]
Barn owl	*Tyto alba*	Nocturnality	Zhang et al. [33]
Chuck-will’s-widow	*Antrostomus carolinensis *(*Caprimulgus carolinensis*)^b^	Nocturnality	Zhang et al. [33]
Great crested grebe	*Podiceps cristatus*	Foot-propelled diving	Zhang et al. [33]
Red-throated loon	*Gavia stellata*	Foot-propelled diving	Zhang et al. [33]
Great cormorant	*Phalacrocorax carbo*	Foot-propelled diving	Zhang et al. [33]
Peregrine falcon	*Falco peregrinus*	Raptorial behavior	Zhan et al. [75]
White-tailed eagle	*Haliaeetus albicilla*	Raptorial behavior	Zhang et al. [33]
Bald eagle	*Haliaeetus leucocephalus*	Raptorial behavior	Zhang et al. [33]
Turkey vulture	*Cathartes aura*	Raptorial behavior	Zhang et al. [33]
Zebra finch	*Taeniopygia guttata*	Related to falcons^a^	Warren et al. [76]
Downy woodpecker	*Dryobates pubescens *(*Picoides pubescens*)^b^	Related to owls^a^	Zhang et al. [33]
Little egret	*Egretta garzetta*	Related to cormorants^a^	Zhang et al. [33]
American flamingo	*Phoenicopterus ruber*	Related to grebes^a^	Zhang et al. [33]
Chicken	*Gallus gallus*	Outgroup^a^	Wong et al. [77]
Ostrich	*Struthio camelus*	Outgroup^a^	Zhang et al. [33]

The “Trait of interest” column shows the convergent trait of each species, except for those marked with “^a^”, which indicate taxa that are closely related to the convergent taxa, or outgroups

^a^ Those species were chosen because they are closely related to species with traits of interest or were used as outgroups in analyses

^b^ The focal species’ old scientific name, which was used in the cited study

**Fig. 2:**
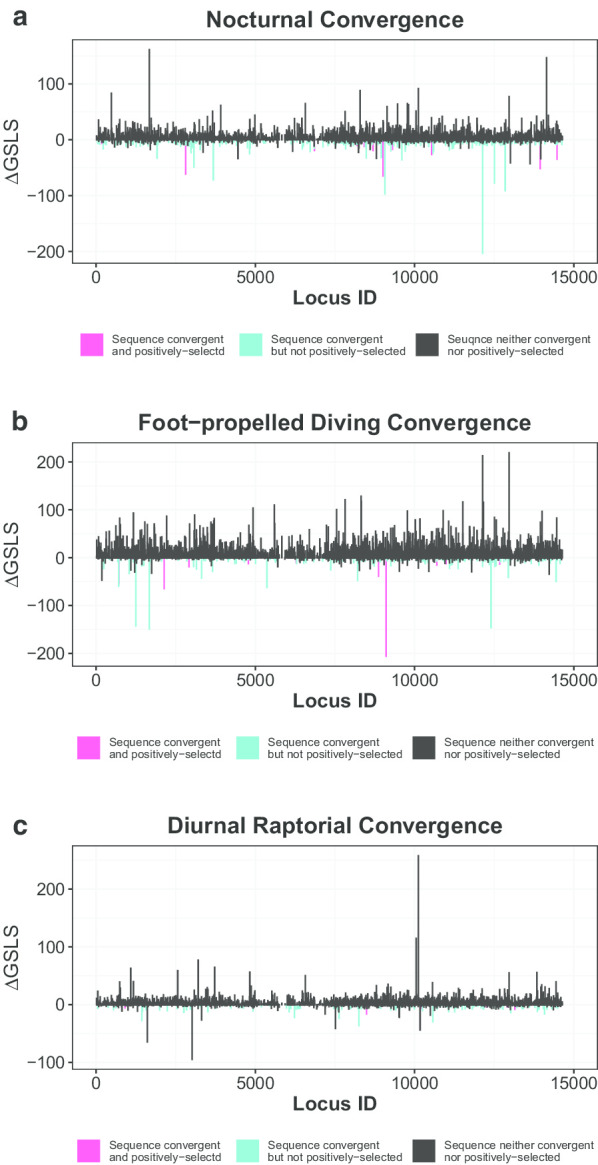
ΔGSLS (gene-specific log-likelihood support) values for **a** H_noc_, **b** H_foot_ and **c** H_rap_ and positive selection signals from 4278 coding genes. The smaller negative ΔGSLS values indicate higher support for H_foot_, H_noc_ or H_rap_ and thus higher levels of sequence convergence. Genes showing significantly smaller ΔGSLS values are in blue, and genes showing both signally smaller ΔGSLS values and significant positive selection signals (i.e., adaptively convergent genes) are in red

We first estimated protein sequence convergent levels between the avian taxa with phenotypic convergence by comparing the likelihoods that the sequences fit to a species tree (used as the null hypothesis, H_0_) and three hypothetical trees that forced the convergent taxa into a monophyletic clade (an step modified from Parker et al. [12]; Fig. 1): (1) H_noc,_ nocturnal convergence between the barn owl (*Tyto alba*) and chuck-will’s-widow (*Antrostomus carolinensis*); (2) H_foot_, foot-propelled diving convergence among the red-throated loon (*Gavia stellate*), great cormorant (*Phalacrocorax carbo*) and great crested grebe (*Podiceps cristatus*); (3) H_rap_, raptorial convergence between the peregrine falcon (*Falco peregrinus*) and three other raptors—the turkey vulture (*Cathartes aura*), white-tailed eagle (*Haliaeetus albicilla*) and bald eagle (*Haliaeetus leucocephalus*). Among the 4278 genes, 352 were found to contain convergent sequences consistent with H_noc_, 206 with H_foot_ and 117 with H_rap_ (i.e., genes with significantly higher likelihoods fitting to the convergent hypotheses than to H_0_; see “Methods” for details; Fig. 2).

We then applied the positive selection test to the above sequence-convergent genes to identify ones contributing to adaptive evolution and thus likely driving convergent phenotypical changes (the second step of the approach). For the sequence-convergent genes, positive selection signals were then detected in 24 genes for nocturnal birds, 13 for foot-propelled diving birds and 6 for diurnal raptors (see “Methods” for details;); we considered these ACGs contributing to the focal phenotypic convergence because they had convergent sequences and were under positive selection in the convergent taxa (Fig. 2 and Additional file 1: Table S1).

### Evolutionary constraints from site mutations to genetic pathways

To gauge genetic constraints across different genetic hierarchical levels, we first focused on constraints below the gene level by estimating the numbers of positively-selected (amino acid) site mutations shared between convergent taxa (but not other study taxa; see “Methods” for details). We only found a few “adaptively convergent site mutations (ACSMs)” in a small part of the ACGs. We identified nine ACSMs in eight (out of 24) nocturnal ACGs, three ACSM in two (out of 13) foot-propelled diving ACGs and one ACSM in one (out of six) diurnal raptorial ACG. To understand whether the observed numbers of ACSMs surpassed the numbers of convergent site mutations that would be independently obtained by lineages under neutral evolution, we calculated locus-wise “neutral values” (see “Methods” for details) against which we compared the observed values. With neutral values summed up by hypotheses, we found that the numbers of observed ACSMs were higher than or close to the neutral expectations (= 3.0440 sites for the nocturnal ACGs, 0.0001 sites for the foot-propelled diving ACGs and 1.0878 sites for the diurnal raptorial ACGs, Additional file 1: Table S2). That is, the ACGs identified by our approach indeed had generally higher levels of sequence convergence than did the neutral expectation. However, given that ACSMs were only found in a few ACGs, divergent amino acid replacements in most ACGs might still cause phenotypes to converge, a finding consistent with previous studies [37]. The results suggest low constraint for phenotypic convergence at the site mutation level.

We then assessed constraints above the level of individual genes by examining whether the ACGs were highly enriched in particular genetic pathways or functional groups. We conducted DAVID [38] analyses and found that none of the ACGs of the three convergent characters showed significant signals of functional enrichment. Furthermore, we conducted STRING analyses [39] and found that these ACGs had no significantly enriched signal for any protein–protein interaction (PPI) or protein functionality. In addition, we conducted the enrichment tests on expanded datasets, including ACGs and sequence-convergent genes that showed a positive selection signal in only one of the lineages with the focal convergent traits (n = 40, 29 and 11 for nocturnal, foot-propelled diving and raptorial convergence, respectively; Additional file 1 Table S3). The DAVID analyses also returned no enrichment signal. The STRING analyses showed no PPI enrichment, but did return a functional enrichment signal in two genes (*PLCG1* and *PLCXD2*; false discovery rate = 0.0029) for the raptorial convergence. However, the enriched term (PLC-like phosphodiesterase, TIM beta/alpha-barrel domain superfamily) is only related to the protein structure, and the two genes share no biological function. Take together, these results indicate low constraint above the gene level for convergent evolution.

**Fig. 3:**
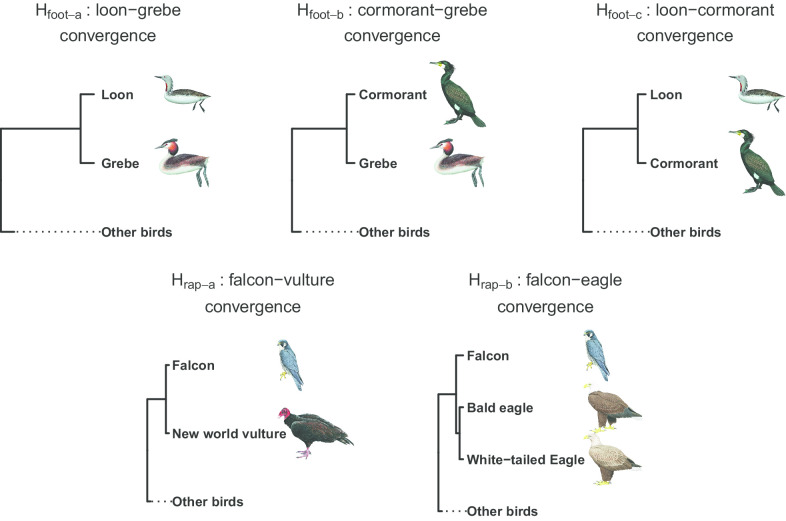
Derived hypotheses for genomic sequence convergence. Three convergence hypotheses (H_foot-a_, H_foot-b_ and H_foot-c_) were derived from the foot-propelled diving convergence hypothesis (H_foot_ in Fig. 1) and two (H_rap-a_ and H_rap-b_) from the raptorial convergence hypothesis (H_rap_ in Fig. 1) (All bird illustrations were reproduced with permission from Lynx Edicions)

**Fig. 4:**
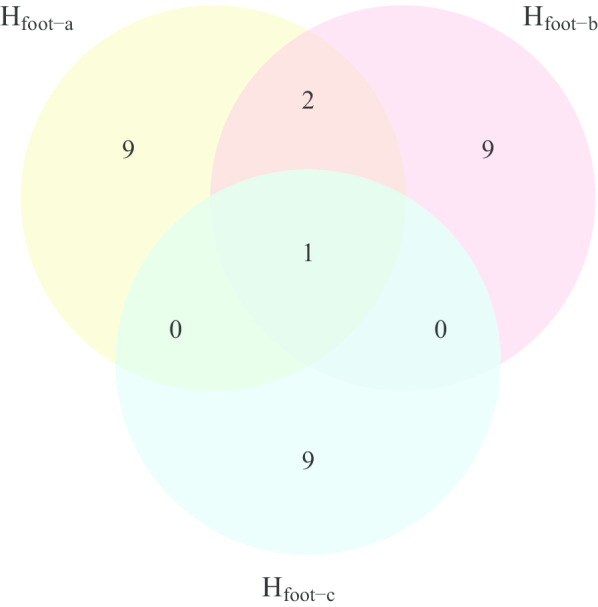
Numbers of adaptively convergent genes that support H_foot-a_, H_foot-b_ and H_foot-c_. The Venn diagram was generated using the R package, VennDiagram [78]

To infer evolutionary constraints at the individual gene level, we formulated two hypothetical trees derived from H_foot_ and H_rap_ to force different convergent pairs to form clades (i.e., H_foot-a_, H_foot-b_, H_rap-a_ and H_rap-b_; Fig. 3; see “Methods” for details). If the ACGs supporting the two derived hypotheses largely overlapped in each case of phenotypic convergence, then the results would suggest strong genetic constraint. For the foot-propelled diving convergence, we found 12 ACGs supporting H_foot-a_ and 12 supporting H_foot-b_ (Fig. 4 and Additional file 1: Table S4). However, only three of these ACGs were found in both of the above lists (Fig. 4 and Additional file 1: Tables S1 and S4). To obtain a neutral expectation for the overlap ratio, we calculated the number of genes with neutrally convergent sequences (i.e., sequence-convergent genes that were not ACGs) to be 82 for H_foot-a_ and 87 for H_foot-b_, and found that 42 of them overlapped. We found that the proportion of overlapping ACGs between H_foot-a_ and H_foot-b_ was not significantly higher than that of the neutrally sequence-convergent genes (p = 0.1299, two-tailed Fisher’s exact test). For the raptorial convergence, we found 16 ACGs supporting H_rap-a_ and 12 supporting H_rap-b_; only one of these was found in both of the above lists (Additional file 1: Tables S1 and S4). The number of neutrally sequence-convergent genes was 130 sequences for H_rap-a_ and 162 for H_rap-b_, and 20 of these overlapped. The proportion of overlapping ACGs between H_rap-a_ and H_rap-b_ was also not significantly higher than that of the neutrally sequence-convergent genes (p = 0.7055, two-tailed Fisher’s exact test). Thus, adaptive selection regarding foot-propelled diving and raptorial behavior did not tend to use the same genes to reach phenotypic convergence between different convergent pairs, suggesting low constraint at the gene level.

### Relationships between phylogenetic distance and molecular convergence

A trend in which more ACGs are shared between the phylogenetically closer taxa than the ecologically closer taxa would suggest that genetic background plays a dominant role in convergent evolution; from there, evolutionary processes and outcomes could be predicted. To determine whether there was a trend in the present study, we formulated a third hypothesis derived from H_foot_ (i.e., H_foot-c_, in which loons and cormorants were forced into a clade; Fig. 3), and found that the number of ACGs shared between loons and cormorants (n = 10) was similar to those between loons and grebes (i.e., H_foot-a_, n = 12; p = 0.67, chi-square test; Fig. 4 and Additional file 1: Table S4). That is, there were not more ACGs associated with convergence between loons and cormorants, which were phylogenetically closer, than such ACGs between loons and grebes, which were more phenotypically similar. Thus, the shared genetic mechanism underlying foot-propelled diving convergence could not be predicted by phylogenetic closeness. In addition, only one ACG overlapped between the two pairs (Fig. 4) and the overlap level was not higher than the neutral expectation (p = 1, two-tailed Fisher’s exact test).

## Discussion

Even though Gould’s [1] theory about replaying the tape of evolutionary history cannot be physically tested, the convergence of lineages that face similar selective forces presents a reasonable analogy [40]. Additionally, in studying one single lineage, it is difficult to remove linage-specific historical signals from genotype–phenotype associations; however this can be overcome by comparing independent lineages with convergent traits [41]. In this study, we applied comparative genomics approaches to three cases of phenotypic convergence in birds to decipher the genetic codes for complex behavioral adaptations. Our results suggest that evolution is subject to low genetic constraint, implying that evolution is unpredictable—at least at the level of avian order.

### Low genetic constraints for avian convergence, from site mutations to genetic pathways

The numbers of convergent genes identified in our study are lower than those of other genome-level studies. This study shows that the convergence of nocturnality, foot-propelled diving and raptorial behavior can be attributed to at least 24, 13 and 6 ACGs, respectively. In contrast, Parker et al. [12] found nearly 200 genes associated with echolocation convergence between bats and dolphins; Chikina et al. [41] identified hundreds of genes associated with convergence in marine mammals. However, it should be noted that our analytical approaches, which apply restrictive statistical criteria to determined ACGs (see “Methods” for details), are not identical to theirs. Furthermore, the targeted phenotypic traits in this and previous studies have different levels of complexity, making a direct comparison unproductive. Nevertheless, the three convergence cases examined in our study suggest that the numbers of ACGs underlying complex life history traits can vary. The relatively low numbers of ACGs found in our study may also imply that these taxa do not extensively share the genetic bases of their convergent phenotypes, a conclusion further supported by our other analyses, discussed below.

Most ACGs identified in this study did not have any ACSMs, and those that did only had one or two. These convergent genes were also barely enriched in any genetic pathways or biological function and showed no significant protein interactions among one another. Given that complex behavioral traits are likely determined by multiple genes associated with particular functional groups [12, 41], the above results imply that the taxa we analyzed might use different genes in their genetic pathways or functional groups to generate similar phenotypic changes. Furthermore, our tests of derived convergence hypotheses (i.e., H_foot-a_, H_foot-b_, H_rap-a_ and H_rap-b_) showed that overlaps in ACGs among pairs of avian orders with the same convergent phenotypes were not different from the neutral expectation, indicating low constraint at the gene level. Therefore, these cases of convergent evolution are subject to low genetic constraints, from site mutations [42], to genes and genetic pathways [43]. The results indicate that the evolutionary history of these convergent traits is unlikely to be predictable from a genomic perspective; this is consistent with the contingent view of evolution proposed by Gould [1] and some recent studies ([42, 44, 45]; but see [9, 14, 40, 46]).

### Selection force surpasses phylogenetic closeness in causing adaptive genes to reoccur in foot-propelled diving convergence

Another way to assess the predictability of evolution is to examine whether more closely related taxa tend to use more similar genetic mechanisms to generate convergent phenotypes [41]—i.e., whether the probability of gene reuse in convergent phenotypic evolution is determined by the initial (genomic) states of focal organisms. Even though phenotypic convergence is often treated as the results of similar selection pressures imposed by shared environmental challenges (i.e., external factors), it can also be attributed to limited genetic variations (i.e., internal factors; [31]). Phylogenetic relationships may impact the availability of shared genetic variations because more closely related taxa have a better chance of sharing genetic variations passed down from their common ancestors and thus use them to generate the same phenotypes [31]. It is also possible that the same amino acid substitutions can have the same function only under similar genomic backgrounds that tend to be found among closely related taxa [42, 47]. If phenotypic convergence is mainly caused by the limit in available genetic variations or similar genomic backgrounds, then we can expect to find more ACGs between closely related taxa than distinct taxa. In contrast, if natural selection is the dominant force driving convergent evolution, then ACGs between distinct taxa should be at least as common as those between closely related taxa [31].

In this study, we found that foot-propelled diving convergent lineages with closer phylogenetic relationship did not share more ACGs. Loons and grebes have high levels of morphological and behavioral similarities, but are distinct lineages [21, 23]; cormorants, another avian lineage characterized by foot-propelled diving behavior, are more closely related but less phenotypically similar to loons than are grebes [19]. Interestingly, our results showed that loons shared a similar number of ACGs with cormorants to they did with grebes, suggesting that adaptive convergence between the foot-propelled diving lineages is not channeled by their initial genomic backgrounds. In addition, our results also showed low ACG overlap between the two convergent hypotheses (Fig. 4), corroborating that the molecular underpinnings of the foot-propelled diving lifestyle are unrepeatable.

Several previous studies reported negative correlations between phylogenetic distance and convergent gene numbers between convergent taxa [40, 47], suggesting evolutionary processes and outcomes predictable. For example, more closely related birds were shown to have a more similar molecular basis for their hemoglobin [42]. However, some suggested that convergent evolution can only be predicted (i.e., subject to strong constraint) when focusing on a suitable phylogenetic scale, in which species share similar genomic backgrounds [48]. Our study provides evidence that natural selection can be as important as or even outweigh phylogenetic closeness, causing the same genes to reoccur in avian orders that diverged up to nearly 65 million years ago [19]. Our results suggest that shared history is not always the predominant forces driving convergent evolution.

Our findings have two opposing implications. On the one hand, evolution in birds is less predictable at phylogenetic levels equal to or higher than the level of order. This is perhaps because the different avian orders have very different genomic backgrounds, but different genotypes can yield almost identical phenotypes [42, 47]. On the other hand, we found 12 ACGs between grebes and loons, meaning that the phylogenetic distinctness between them still does not completely preclude the possibility that shared genes are responsible for their phenotypic convergence. Even if we attribute convergent evolution to natural selection, it cannot completely rule out the possible impact of constraint imposed by shared genomic backgrounds [49]. Thus, a dichotomous view of convergent evolution’s driver as either natural selection or phylogenetic closeness may not be justified [31]. Essentially, natural selection can only act on genetic variation that is available [3, 40].

### Functional interpretation of ACGs and the genotype–phenotype relationship

Our approach to comparing genomes based on convergent taxa is a promising one for establishing genotype–phenotype relationships, a central issue in evolutionary biology. In the past, searching for candidate genes for phenotypic changes has relied on genes with known functions in model species [46] or genomic data from multiple populations with divergent traits [47]. Now, the increasing number of published genomes of species with convergent phenotypic traits has become an alternative resource for finding candidate genes for phenotypic changes. Here, we identify 43 candidate genes for future functional examination that are strongly associated with one of the three convergent evolution cases (Additional file 1: Table S1).

Owls and nightjars have unique, nocturnal lifestyles that might be explained by a nocturnal ACG, *GRIN1*, which is central in the circadian photic entrainment pathway [50]. Owls and nightjars have relatively large orbits for visual acuity at night [51]; this might be associated with *INHBA*, mutations of which result in enophthalmos, a condition in humans in which the orbits are enlarged [52]. Foot-propelled diving birds have morphologically and physiologically adapted to aquatic environments. For instance, auditory senses might be crucial for diving birds because dim light in deep water may obscure their vision for hunting; the great cormorant was found to have acute underwater hearing comparable to seals and toothed whales [53]. A foot-propelled diving ACG, *KCNC1*, which may impact auditory neuron functioning in mice, may allow these birds to acutely hear underwater [54]. Diurnal raptors are strong predators that often use their feet to grasp preys. Although the connection between their grasping behavior and muscular or neuronal adaptation remains unclear, one raptorial ACG, *CREBRF*, which is associated with the hind limb grasping behavior of mice [55], might contribute to this trait in raptors.

The ACGs that we found to be associated with circadian rhythms in nocturnal birds, auditory adaptation in foot-propelled diving birds and limb grasping in raptors may have medical applications. These avian ACGs are associated with relevant traits in mice or humans, suggesting that birds and mammals could use the same genes to generate convergent traits. Thus, the genotype–phenotype relationships revealed in birds may provide insights to genetic disorders in humans. A better understanding of these ACGs will grant us a better ability to detect people carrying genetic variations associated with circadian rhythm, auditory or muscle problems.

## Conclusions

Our genome-wise analyses consistently identified low genetic constraints in three cases of phenotypic convergence—nocturnality, raptorial behavior and foot-propelled diving—across avian orders. The ACGs that contributed to the focal phenotypic convergence were examined from site mutation to gene and functional pathway levels. We observed (1) few ACSMs among focal taxa, (2) low levels of ACG overlap between taxon pairs with the same convergent phenotypes, and (3) no functional pathways enriched with ACGs; these observations indicate that the focal taxa use largely different molecular bases to achieve phenotypic convergence. The results suggest low genetic constraints across multiple genetic levels. We also found that the frequency of gene reuse in convergent phenotypic evolution is not determined by the initial genomic background of convergent taxa. Taken together, our findings suggest that convergent evolution among avian orders is unpredictable at the molecular level.

While some studies have addressed the genetic basis of convergent evolution using only a few genes associated with particular physiological function [11, 42, 56], our genome-wide assessment of complex traits provides a broad view to explicitly test evolutionary hypotheses such as genetic constraint in phenotypic traits. Our study also demonstrates that genome-wide comparisons among species with phenotypic convergence facilitate the identification of new genes underlying phenotypic traits for non-model species; this practice will become feasible in diverse taxa as genomic data is accumulating rapidly. Although the ACGs identified in this study require further functional tests, they may have medical applications or become new genetic models for functional trait evolution.

## Methods

### Data collection

The coding DNA sequences and protein sequences of 8295 orthologous genes from 48 avian species genomes were obtained from an avian genomic project dataset on the GigaScience Database (https://gigadb.org/dataset/101000; [17, 33, 34]). For the detailed methods used to identify and align the orthologous genes, please refer to the two original studies [17, 33]. In brief, the authors identified orthologous genes across the 48 avian species genomes based on sequence identity to the chicken, zebra finch and human genomes [33]. Firstly, Zhang et al. [33] used synteny in the chicken-zebra finch genome alignment from UCSC [57] to generate 12,484 orthologous genes between the two species. The protein sequences of these orthologous genes were blasted against the human genome. For each pair of chicken-zebra finch orthologous genes, they used the one with the better alignment rate as the reference gene for homology-based gene prediction in the 48 avian genomes; they then generated 13,000–18,000 predicted coding genes. Jarvis et al. [17] re-annotated the orthologous genes in the chick genome based on the zebra finch ones using Genewise [58] and vice versa. The re-annotated reference dataset was used to generate 8295 orthologous genes across the 48 avian genomes based on protein similarity, gene synteny and genome synteny. Jarvis et al. [17] then performed the alignment of orthologous genes using SATé [59] and Prank [60] for the first round and SATé and MAFET [61] for the second round. They used protein sequences to build the alignment and then translated them back to DNA sequences using a custom Perl script.

In this study, we extracted the DNA and protein sequences of 4278 genes from the above alignment dataset, which contained entire or partial sequences for all 16 study species—red-throated loon (*Gavia stellate*, abbreviated as GAVST), great cormorant (*Phalacrocorax carbo*, PHACA), great crested grebe (*Podiceps cristatus*, PODCR), chuck-will’s-widow (*Antrostomus carolinensis*; older name: *Caprimulgus carolinensis*, CAPCA), barn owl (*Tyto alba*, TYTAL), chicken (*Gallus gallus*, GALGA), ostrich (*Struthio camelus*, STRCA), zebra finch (*Taeniopygia guttata*, TAEGU), turkey vulture (*Cathartes aura*, CATAU), peregrine falcon (*Falco peregrinus*, FALPE), white-tailed eagle (*Haliaeetus albicilla*, HALAL), bald eagle (*Haliaeetus leucocephalus*, HALLE), Anna’s hummingbird (*Calypte anna*, CALAN), little egret (*Egretta garzetta*, EGRGA), American flamingo (*Phoenicopterus ruber*, PHORU) and downy woodpecker (*Dryobates pubescens*; older name: *Picoides pubescens*, PICPU; Table 1)—using a custom Perl script. Only 16 of the 48 species published by Jarvis et al. [17] were chosen for analyses because (1) these species were enough to test the proposed hypotheses (see below for details), (2) more species would lead to fewer genes that could be aligned due to missing data, and (3) we aimed to reduce biases introduced by species that show partial levels of the focal characteristics, such as semi-nocturnality [62] or diving adaptation. We removed gaps, ambiguous sites and in-frame stop codons from the coding sequences using PAL2NAL [63].

### Two-step ACG identification approach (step one): testing genomic sequence convergence

We developed a two-step approach to identify genes that contribute to the phenotypic convergence of the study taxa. The first step was to find genes with sequences that were convergent among phenotypically convergent taxa (referred to as sequence-convergent genes). The second step was to identify sequence-convergent genes that showed positive selection signals in all of the focal convergent taxa; the genes identified were referred to as “adaptively convergent genes” (ACGs). The first step was modified from Parker et al.’s [12] site-specific log-likelihood support (SSLS) approach. Because the SSLS approach has been criticized by two studies [35, 36] for failing to establish reasonable null models, we modified this step to address the critiques (see below for details) and added the second step to increase the chance of identifying genes causing phenotypic convergence, analogous to Thomas and Hahn’s [35] approach.

For the first step, we estimated the level of gene-wise protein sequence convergence for avian orders with phenotypic convergence by testing three convergent hypotheses (Fig. 1)—(1) H_noc_, nocturnal convergence between one Caprimulgiformes species (CAPCA) and one Strigiformes species (TYTAL); (2) H_foot_, foot-propelled diving convergence among three water birds in Gaviiformes, Suliformes and Podicipediformes (i.e., GAVST, PHACA and PODCR, respectively); (3) H_rap_, raptorial convergence between three species from Accipitriformes/Cathartiformes (HALAL, HALLE and CATAU) and one from Falconiformes (FALPE). A species tree of the 16 study species modified from Jarvis et al. [17] was used as the null hypothesis (H_0_). For each convergent hypothesis, we restricted the hypothetical tree topology by forcing each convergent group into a monophyly. When moving one or more of the focal species in the phylogenetic tree to generate a hypothetical convergent clade, we consistently made this clade far from, rather than close to, STRCA (i.e., the first lineage to branch out in the avian phylogeny). We used PAML 4.9 [64] to estimate gene-specific log-likelihood support (GSLS), which measured how well each gene’s protein sequences fit to the species tree (H_0_) and hypothetical trees (H_rap_, H_foot_ and H_noc_). The GSLS approach was modified from Parker et al.’s [12] SSLS approach to estimate a likelihood for each gene instead of each site. The differences between the GSLS values of H_0_ and alternative convergent hypotheses (ΔGSLS(H_n_) = GSLS(H_0_) − GSLS(H_n_)) were used to test whether the targeted genes showed sequence convergence among the focal convergent taxa. Lower negative ΔGSLS values indicated higher levels of support for sequence convergence.

Studies [35, 36] have criticized Parker et al.’s [12] SSLS approach for failing to establish reasonable null models for ΔSSLS values by taking into account the signals from non-convergent pairs. To address the critiques, we conducted simulations based on a branch-moving approach to generate null models for assessing the statistical significance of empirical ΔGSLS values. This approach aimed to control random chances of sequence convergence among the study taxa. We used Mesquite v3.5 [65] to randomly switch one and two branches in the species tree (H_0_) to generate simulated trees corresponding to the numbers of branch switching for H_noc_/H_rap_ and H_foot_, respectively. That is, we simulated all possible restricted monophylies for any two or three study taxa, both convergent and non-convergent ones, against the species tree with the same amount of phylogenetic distortion from H_0_ to H_noc_/H_rap_ or H_foot_. In total, there were 663 and 18,570 possible tree topologies generated in the one- and two-branch-moved simulations, respectively. Given the large number of possible topologies in the latter, we randomly sampled 1000 topologies from it for subsequent analyses (i.e., ΔGSLS calculation) to make computational time more manageable. The ΔGSLS values of the 663 one-branch-moved and 1000 two-branch-moved trees were estimated for each of the 4278 examined genes. That is, two sets of simulated ΔGSLS values (one for 663 one-branch-moved trees v.s. the species tree, and the other for 1000 two-branch-moved trees v.s. the species tree) for each gene were generated and used as the null distributions of ΔGSLS values.

For the one-branch-moved simulation (corresponding to H_noc_ and H_rap_), we calculated the 5th percentile of the simulated ΔGSLS distribution (i.e., the lower boundary of its one-sided 95% confidence interval, CI), and used it as a threshold value to determine whether the empirical ΔGSLS value was significantly low. The procedures were conducted separately for each of the 4278 examined genes. Only genes with an empirical ΔGSLS value smaller than their corresponding threshold values were treated as having significantly convergent sequences (i.e., sequence-convergent genes) for the focal convergence hypotheses.

For the two-branch-moved simulation (corresponding to H_foot_), given that we could not estimate ΔGSLS values for all possible simulated trees due to extremely long computing time, we used a bootstrapping method to estimate the 5th percentile of its complete ΔGSLS distribution. We bootstrapped the ΔGSLS values from the simulated datasets (i.e., randomly sampled them with replacement to their original sample size, 1000) to obtain one bootstrap resample of ΔGSLS values. We then calculated the 5th percentile of the bootstrap resample. We repeated the bootstrap procedure 1000 times and calculated the mean of the 1000 bootstrap 5th percentiles, which was then used it as a threshold value to determine the statistical significance of the empirical ΔGSLS value. The procedures were conducted separately for each of the 4278 examined genes. To confirm that this bootstrap approach could generate estimated 5th percentiles close to true values, we also applied the same procedure to the one-branch-moved simulated dataset, for which we knew the true 5th percentile of the complete ΔGSLS distribution for every examined gene. For the one-branch-moved dataset, we found that (1) true 5th percentiles were always within the 95% CIs of the bootstrap 5th percentiles for all genes, (2) the difference between the true and estimated 5th percentiles (i.e., the means of 1000 bootstrap 5th percentiles) only averaged 5% of the full ranges of the bootstrap values, and (3) genes that were concluded to be significantly sequence-convergent based on the estimated 5th percentiles were the same as those based the true 5th percentiles. The above results suggest that this bootstrap approach was reliable. We used the R package boot [66] to conduct the bootstrap analyses.

### Two-step ACG identification approach (step two): testing positive selection signal

For the second step, we further examined whether the genes with significant sequence convergence between phenotypically convergent orders (i.e., with significantly low ΔGSLS values; sequence-convergent genes) were also under positive selection. Only genes with significant sequence convergence and positive selection signals in phenotypically convergent taxa were treated as contributing to the convergence of the focal traits and referred to as ACGs. The selection tests were based on the dN/dS ratios (ω; dN, non-synonymous substitutions; dS, synonymous substitutions) of DNA sequences, estimated by PAML. We conducted the branch-site models to compare the likelihood of the species tree with pre-specified branches under positive selection (i.e., foreground; the model A) with that of the same phylogeny with ω_2_ fixed to 1 (i.e., foreground as neutral; the corresponding null model). The foreground taxa were the focal taxa for each convergence hypothesis. We used the likelihood ratio test to examine whether the differences between the likelihood of these two models were large enough to conclude that the genes of the focal taxa have experienced positive selection. A significance level of 0.05 for the likelihood ratio corresponded to a threshold χ^2^ value of 3.84 [64].

To rule out the possibility that genes showing positive selection signals in the foreground taxa were also subject to selection in some of the background taxa (i.e., the non-focal taxa), we applied the site models to a species tree that excluded the focal taxa. We compared the likelihood of the M1a model (as a neutral model) against that of the M2a model (as a positive selection model) for genes with the significant results of the branch-site model A. A significance level of 0.05 for the likelihood ratio for the site models corresponded to a threshold χ^2^ value of 5.99 [64]. If genes showed significant results for both branch-site and site models, then they were excluded from subsequent analyses; one to five sequence-convergent genes significantly supported both models for each convergence hypothesis. Therefore, we focused on genes that showed positive selection signals in the focal taxa but not other taxa.

Ones might argue that our approach risks misidentifying AGCs. That is mainly because the results of the ΔGSLS test (i.e., sequence-convergent genes identified in step one) might also reflect phylogenetic discordance between the species tree and gene trees caused by evolutionary events other than adaptive convergence, including paralogs of genes, introgression or incomplete lineage sorting [67]. We argue that these confounding factors had limited impact on our final results because (1) the genes we examined were orthologs [17], (2) the second step of our approach could largely exclude sequence-convergent genes with no selection signal (i.e., non-adaptive genes) in the focal taxa, and (3) even if, in some cases, sequence-convergent genes caused by incomplete lineage sorting or introgression might not be excluded by the selection tests, their impact on our conclusion should be relatively minor (see below for details).

Here we used the species tree for the positive selection tests because selection may bias the estimation of gene trees, and thus the species tree better represents the evolutionary history of adaptive genes. For example, convergent taxa could be a monophyly in a reconstructed gene tree when selection independently retained convergent mutations in distinct lineages (i.e., more shared mutations do not essentially mean a closer phylogenetic relationship between two taxa in a gene’s history) [e.g., see Ref 13]. Such incorrectly reconstructed gene trees could lead positive selection tests to yield false-negative results due to an underestimated number of substitutions along the foreground branches. On the other hand, we acknowledged that use of the species tree, instead of gene trees, may potentially lead to false-positive selection test results if taxa with phenotypic convergence form a monophyly in the latter due to incomplete lineage sorting or introgression. This is because the former might assume more substitutions along foreground branches than the latter [68]. To assess the prevalence of this condition, we first reconstructed maximum likelihood gene trees for the 43ACGs identified in this study using IQ-TREE v.1.6.1 [69, 70] and found that the convergent taxa formed a monophyly in only around 1/6 of the trees (6 out 24 ACGs, 2 out of 13 ACGs and 1 out of 6 ACGs for nocturnal, foot-propelled diving and raptorial convergence, respectively) (Additional file 1: Fig. S1). Thus, false-positive results should be relatively rare in selection tests. Even if there were any false-positive results, our conclusion that these convergent phenotypes have low genetic constraint would remain unchanged because the ACGs could only decrease in number, suggesting an even lower level of constraint.

Secondly, if incomplete lineage sorting erroneously increased the identification of sequence-convergent genes and then caused false-positive results in the selection tests, we would expect the biased effect to be worse in closely related than distinct convergent taxa. That is because the coalescence time between closely related taxa is shorter than that between distinct taxa, leading to higher chances of incomplete lineage sorting. If so, the number of ACGs supporting H_foot-c_ should be higher than that of H_foot-a_ because the former forced a monophyly for a pair of more closely related taxa (loon-cormorant) than the latter (loon-grebe; see the section “Relationships between phylogenetic distance and molecular convergence in foot-propelled birds” for the details of the two hypotheses). However, the two hypotheses returned similar numbers of ACGs, suggesting weak impact of incomplete lineage sorting on identifying ACGs.

Thirdly, the sequences we used in this study were amino acid or coding regions, which might be less subject to incomplete lineage sorting than non-coding regions due to smaller effective population sizes [71]. Overall, we believe that the biased effect of incomplete lineage sorting or introgression on our results was relatively minor and did not affect our conclusion.

### Evolutionary constraint below, above and at the gene level

We assessed the evolutionary constraints below the gene level based on the numbers of shared (amino acid) site mutations between convergent taxa. If the ACGs had a large number of convergent site mutations with positive selection signals, then the focal phenotypic convergence was likely caused by a high degree of convergence at those site mutations, suggesting strong evolutionary constraint below the level of individual genes. In contrast, a low number of convergent site mutations would suggest low evolutionary constraint below the gene level.

We used the Bayes empirical Bayes (BEB) approach implemented in PAML to calculate the posterior probabilities of site-wise positive selection signals for the ACGs identified in the last section. If the posterior probability that ω > 1 at one site was greater than 95% (i.e., significant positive selection signal), then we further examined whether its sequences converged in the focal taxa but not others using Jalview [72]. We referred to sites with significant BEB signals of positive selection and convergent sequences among the focal taxa as “adaptively convergent site mutations” (ACSMs). These ACSMs were assumed to contribute to the corresponding types of phenotypic convergence.

For each ACG, we further calculated the expected number of convergent site mutations (*M*) that could arise through neutral evolution and compared this to the number of identified ACSMs. We used this test to examine whether the ACGs identified by our approach had higher sequence convergent levels than the neutral expectation. For the nocturnal and the raptorial convergent hypotheses, such *M* values were each calculated as (*D* − *P*)/20 × *L*, where *D*, *P* and *L* were mean pairwise Poisson-correction distance, mean pairwise p distance and locus length, respectively; thus, *M* represented the number of multiple-substitution sites between two distinct lineages that each such site used the same amino acid residue among a total of 20 options. For the foot-propelled diving hypothesis, we calculated each *M* value as [(*D* − *P*)/20]^2^ × *L* (the number of multiple-substitution sites between three distinct lineages that each such site used the same amino acid residue). *D* and *P* were calculated in MEGA X [73] with pairwise deletions for gaps and a uniform rate among sites, and were computed only between non-convergent birds depending on the hypothesis considered, so as to avoid any non-neutral signals. Provided that, for each ACG, both observed (ACSM number) and expected (*M*) values were small and thus subject to stochastic errors, we summed up values over genes based on hypotheses to make comparisons (Additional file 1: Table S2).

We also assumed that more ACGs identified along genetic pathways meant that the focal taxa experienced stronger constraint above the gene level during their convergent evolution. Thus, we assessed the extent to which the focal taxa shared ACGs along genetic pathways or within functional groups to generate their convergent traits by examining the functional relationship among the ACGs. We did this by testing for functional enrichment in gene ontology (GO) terms, KEGG pathways, UP_TISSUE and INTERPRO databases for the ACGs using DAVID 6.8 (https://david.ncifcrf.gov/, [38]) We applied the Fisher’s exact test with the Benjamini–Hochberg correction [74]. We also used STRING 11.0 (https://string-db.org/, [39]) to assess over-representation in protein–protein interactions (PPI) and functional enrichment for the ACGs that imply functional partnerships among them. In addition, we also considered genes that contributed to adaptive convergence in only one of the focal lineages; the analyses might facilitate the identification of overlapped pathways and functions among different adaptive genes, although such involved analyses might somewhat overestimate the overlap level due to the possibility of including genes that did not cause the convergent traits. In practice, we applied the DAVID and STRING tests to datasets containing ACGs and sequence-convergent genes with positive selection signals in one (set as the foreground of branch-site models for the PAML analyses) of the convergent lineages. Both DAVID and STRING used all of the 4278 genes as a background list.

We further formulated two hypotheses derived from H_foot_ and H_rap_ (Fig. 3) to examine the evolutionary constraint at the gene level. In one derived hypothesis (H_foot-a_) from H_foot_, we forced GAVST and PODCR together in a clade and excluded PHACA; in the other hypothesis (H_foot-b_), we forced PHACA and PODCR into a clade, without GAVST (Fig. 3). In one derived hypothesis (H_rap-a_) from H_rap_, we forced FALPE and CATAU into a clade, not including HALAL and HALLE; in the other hypothesis (H_rap-b_), we forced FALPE, HALAL and HALLE into a clade, without CATAU (Fig. 3). We performed sequence convergence hypothesis tests for H_foot-a_, H_foot-b_, H_rap-a_ and H_rap-b_ against H_0_, respectively. We applied the Benjamini–Hochburg correction to the multiple derived hypotheses that focused on the same phenotypic convergence. First, we calculated the percentile rank (assuming *X*th percentile) of the derived hypothesis’ ΔGSLS value in the one-branch-moved simulated values (for 663 possible trees) for each of the 4278 examined genes. We then corrected the *X* values with the Benjamini–Hochburg procedure using the R function “p.adjust” and concluded that genes have significantly convergent sequences (i.e., sequence-convergent genes) only if their adjusted *X* values < 5. Finally, we conducted positive selection tests for the sequence-convergent genes to identify ACGs, as what we did for the original hypotheses. We compared the ACGs supporting H_foot-a_ with those supporting H_foot-b_, and the ACGs supporting H_rap-a_ with those supporting H_rap-b_ to measure the levels of shared ACGs between pairs of convergent lineages. We then compared the overlap proportion of ACGs with that of neutrally sequence-convergent genes (i.e., sequence-convergent genes that were not ACGs) between H_foot-a_ and H_foot-b_ or between H_rap-a_ and H_rap-b_ using the two-tailed Fisher’s exact test. If the ACGs supporting the two derived hypotheses had a significantly higher overlap level than did that of the neutrally sequence-convergent genes, then the results suggested that natural selection tended to use the same genes to reach to phenotypic convergence; such results indicate strong evolutionary constraint at the gene level.

### Relationships between phylogenetic distance and molecular convergence in foot-propelled birds

We used the foot-propelled diving birds as a system to examine the relative effects of natural selection and phylogenetic closeness on genes that make up convergent phenotypes. We formulated a third hypothesis derived from H_foot_ (H_foot-c_; the first two were H_foot-a_ and H_foot-b_, shown above). In H_foot-c_, we forced GAVST and PHACA into a clade, without PODCR (Fig. 3). We performed the sequence convergence hypothesis tests with the Benjamini–Hochburg correction (see previous section) and then conducted positive selection tests to examine whether there were more ACGs supporting H_foot-c_ than H_foot-a_ (which forced GAVST and PODCR into a clade). By doing so, we could determine whether the level of genetic convergence was higher in the phylogenetically closer pair (GAVST and PHACA) or the ecologically closer pair (GAVST and PODCR). This examination might illuminate the predictability of evolution. If the initial background of convergent taxa could predict the convergent level of their genetic underpinnings, then we would expect the phylogenetically closer taxa to share more ACGs than the ecologically closer taxa.

## Supplementary information

**Additional file 1: Table S1.** Adaptively convergent genes for nocturnal, foot-propelled diving or raptorial life history traits.** Table S2.** The total number of adaptively convergent site mutations (ACSMs) in adaptively convergent genes (ACGs) and the number of expected point mutations under neutral selection.** Table S3.** Sequence-convergent genes that show positive selection signals in parts or all of the foreground branches regarding nocturnal, foot-propelled diving or raptorial life history traits.** Table S4.** Adaptively convergent genes supporting for one of the derived convergence hypotheses (H_foot-a_, H_foot-b_, H_foot-c_, H_rap-a_ and H_rap-b_, see Fig. 3).** Figure S1.** Maximum likelihood gene trees of 43 adaptively convergent genes, supporting one of the tree convergent hypotheses – nocturnality (Hnoc), foot-propelled diving (H_foot_) and raptorial behaviors (H_rap_)

## Data Availability

The analyzed genome sequences are publicly available and were downloaded from the GigaScience Database (https://gigadb.org/dataset/101000; [34]). The DNA and protein sequence alignments and in-house developed codes used in this study are available in the FigShare repository, 10.6084/m9.figshare.13175135.

## Abbreviations

ACG: Adaptively convergent gene
H_0_: Null hypothesis of phylogeny
H_noc_: Hypothetical phylogeny for nocturnal convergence
H_foot_: Hypothetical phylogeny for foot-propelled diving convergence
H_rap_: Hypothetical phylogeny for raptorial convergence
ACSM: Adaptively convergent site mutation
*PLCG1*: Phospholipase C Gamma 1
*PLCXD2*: Phosphatidylinositol Specific Phospholipase C X Domain Containing 2
H_foot-a_: Derived hypothetical phylogeny for foot-propelled diving convergence, type a
H_foot-b_: Derived hypothetical phylogeny for foot-propelled diving convergence, type b
H_foot-c_: Derived hypothetical phylogeny for foot-propelled diving convergence, type c
H_rap-a_: Derived hypothetical phylogeny for raptorial convergence, type a
H_rap-b_: Derived hypothetical phylogeny for raptorial convergence, type b
*GRIN1*: Glutamate Ionotropic Receptor NMDA Type Subunit 1
*INHBA*: Inhibin Subunit Beta A
*KCNC1*: Potassium Voltage-Gated Channel Subfamily C Member 1
*CREBRF*: CREB3 Regulatory Factor
UCSC: University of California Santa Cruz Genome Brower database
GAVST: *Gavia stellate*
PHACA: *Phalacrocorax carbo*
PODCR: *Podiceps cristatus*
CAPCA: *Caprimulgus carolinensis*
TYTAL: *Tyto alba*
GALGA: *Gallus gallus*
STRCA: *Struthio camelus*
TAEGU: *Taeniopygia guttata*
CATAU: *Cathartes aura*
FALPE: *Falco peregrinus*
HALAL: *Haliaeetus albicilla*
HALLE: *Haliaeetus leucocephalus*
CALAN: *Calypte anna*
EGRGA: *Egretta garzetta*
PHORU: *Phoenicopterus ruber*
PICPU: *Picoides pubescens*
SSLS: Site-specific log-likelihood support
GSLS: Gene-specific log-likelihood support
BEB: Bayes empirical Bayes
*M*: Expected number of convergent site mutations
*D*: Poisson-correction distance
*P*: Mean pairwise p distance
*L*: Locus length
