# Supplementary material for: Avian phenotypic convergence is subject to low genetic constraints based on genomic evidence
Source: BMC Evol Biol. 2020 Nov 7;20:147. doi: 10.1186/s12862-020-01711-7 (PMC7648321; doi:10.1186/s12862-020-01711-7)
Supplement: Supplementary file 1 — Additional file 1: Table S1. Adaptively convergent genes for nocturnal, foot-propelled diving or raptorial life history traits. Table S2. The total number of adaptively convergent site mutations (ACSMs) in adaptively convergent genes (ACGs) and the number of expected point mutations under neutral selection. Table S3. Sequence-convergent genes that show positive selection signals in parts or all of the foreground branches regarding nocturnal, foot-propelled diving or raptorial life history traits. Table S4. Adaptively convergent genes supporting for one of the derived convergence hypotheses (Hfoot-a, Hfoot-b, Hfoot-c, Hrap-a and Hrap-b, see Fig. 3). Figure S1. Maximum likelihood gene trees of 43 adaptively convergent genes, supporting one of the tree convergent hypotheses – nocturnality (Hnoc), foot-propelled diving (Hfoot) and raptorial behaviors (Hrap) [file 12862_2020_1711_MOESM1_ESM.docx]

**Title: Avian phenotypic convergence is subject to low genetic constraints based on genomic evidence**

Yu-Chi Chen^1^, Hao-Chih Kuo^1^, Wen-Sui Lo^2^ and Chih-Ming Hung^1^*

^1^ Biodiversity Research Center, Academia Sinica, Taipei, Taiwan

^2^ Department of Evolutionary Biology, Max Planck Institute for Developmental Biology, Tübingen, Germany

* Corresponding author: Chih-Ming Hung, [cmhung@gate.sinica.edu.tw](mailto:cmhung@gate.sinica.edu.tw) (C.-M. Hung)

**Additional information**

Additional Tables S1 - S4

Additional Figure S1

**Table S1.** Adaptively convergent genes for nocturnal, foot-propelled diving or raptorial life history traits. Locus numbers correspond to the numerical IDs of the aligned gene datasets (48_birds_gene_alignment) of Zhang et al. [33], downloaded from the GigagScience Database (http://gigadb.org/dataset/101000). The ENSEMBL transcript, gene IDs and gene names come from the chick (*Gallus gallus*).

| Locus | ENSEMBL_Transcript_ID | ENSEMBL_GENE_ID | Full name | | Abbr. name |
| --- | --- | --- | --- | --- | --- |
| Nocturnal convergence (H_noc_) | | | | | |
| 175 | ENSGALT00000000849 | ENSGALG00000000607 | G protein-coupled receptor 37 like 1 | GPR37L1 | |
| 994 | ENSGALT00000004933 | ENSGALG00000003120 | nuclear receptor corepressor 2 | NCOR2 | |
| 2384 | ENSGALT00000012177 | ENSGALG00000007528 | transcription elongation regulator 1 | TCERG1 | |
| 2812 | ENSGALT00000014471 | ENSGALG00000008898 | glutamate receptor, ionotropic, N-methyl D-aspartate 1 | GRIN1 | |
| 5023 | ENSGALT00000027621 | ENSGALG00000017098 | general transcription factor IIIA | GTF3A | |
| 6857 | ENSGALT00000040435 | ENSGALG00000001085 | adhesion G protein-coupled receptor G1 | ADGRG1 | |
| 7499 | ENSGALT00000002308 | ENSGALG00000001518 | zinc finger protein 750 | ZNF750 | |
| 8089 | ENSGALT00000005307 | ENSGALG00000003355 | NOP16 nucleolar protein | NOP16 | |
| 8190 | ENSGALT00000005786 | ENSGALG00000003651 | 5'-nucleotidase, cytosolic IIIB | NT5C3B | |
| 8420 | ENSGALT00000006803 | ENSGALG00000004279 | galectin-related inter-fiber protein | GRIFIN | |
| 8613 | ENSGALT00000007652 | ENSGALG00000004795 | - | - | |
| 8696 | ENSGALT00000008126 | ENSGALG00000005070 | adaptor-related protein complex 3, mu 1 subunit | AP3M1 | |
| 8704 | ENSGALT00000008165 | ENSGALG00000005092 | histone deacetylase 11 | HDAC11 | |
| 8758 | ENSGALT00000008419 | ENSGALG00000005241 | dynein, cytoplasmic 1, light intermediate chain 2 | DYNC1LI2 | |
| 8813 | ENSGALT00000008678 | ENSGALG00000005403 | dehydrogenase/reductase (SDR family) member 11 | DHRS11 | |
| 9012 | ENSGALT00000009581 | ENSGALG00000005958 | tripartite motif containing 66 | TRIM66 | |
| 9065 | ENSGALT00000009815 | ENSGALG00000006083 | lamin-L(III)-like | LMINA | |
| 9310 | ENSGALT00000010951 | ENSGALG00000006769 | Kin17 DNA and RNA binding protein | KIN | |
| 10536 | ENSGALT00000016557 | ENSGALG00000010186 | calpain 11 | CAPN11 | |
| 11180 | ENSGALT00000019496 | ENSGALG00000011952 | solute carrier family 25 member 38 | SLC25A38 | |
| 12100 | ENSGALT00000024177 | ENSGALG00000014989 | S100 calcium binding protein Z | S100Z | |
| 13780 | ENSGALT00000036436 | ENSGALG00000017152 | - | - | |
| 13944 | ENSGALT00000037439 | ENSGALG00000012327 | inhibin, beta A | INHBA | |
| 14475 | ENSGALT00000040340 | ENSGALG00000007889 | Coiled-coil domain containing 85A | CCDC85A | |
| Foot propelled-diving convergence (H_foot_) | | | | | |
| 722 | ENSGALT00000003553 | ENSGALG00000002266 | oxoglutarate dehydrogenase-like | OGDHL | |
| 1152 | ENSGALT00000005664 | ENSGALG00000003578 | fibronectin 1 | FN1 | |
| 2138 | ENSGALT00000010970 | ENSGALG00000006782 | mitochondrial ribosomal protein L46 | MRPL46 | |
| 2917 | ENSGALT00000015069 | ENSGALG00000009258 | KIAA1598-like | LOC423919 | |
| 4782 | ENSGALT00000026329 | ENSGALG00000016320 | F-box protein 9 | FBXO9 | |
| 5339 | ENSGALT00000031453 | ENSGALG00000000302 | troponin T type 2 (cardiac) | TNNT2 | |
| 6284 | ENSGALT00000038066 | ENSGALG00000011566 | glycophorin C (Gerbich blood group) | GYPC | |
| 8871 | ENSGALT00000008906 | ENSGALG00000005552 | polypyrimidine tract binding protein 2 | PTBP2 | |
| 9107 | ENSGALT00000010037 | ENSGALG00000006220 | potassium voltage-gated channel, Shaw-related subfamily, member 1 | KCNC1 | |
| 10699 | ENSGALT00000017326 | ENSGALG00000010652 | sterol carrier protein 2 | SCP2 | |
| 10986 | ENSGALT00000018629 | ENSGALG00000011419 | Dual specificity protein phosphatase 4 | DUSP4 | |
| 12677 | ENSGALT00000026366 | ENSGALG00000016347 | apolipoprotein O | APOO | |
| 12978 | ENSGALT00000027620 | ENSGALG00000017097 | mitochondrial translational initiation factor 3 | MTIF3 | |
| Diurnal raptorial convergence (H_rap_) | | | | | |
| 908 | ENSGALG00000002878 | ENSGALT00000004538 | CREB3 regulatory factor | CREBRF | |
| 8493 | ENSGALG00000004475 | ENSGALT00000007125 | - | - | |
| 10216 | ENSGALG00000009370 | ENSGALT00000015264 | - | - | |
| 12265 | ENSGALG00000015375 | ENSGALT00000024806 | phosphatidylinositol-specific phospholipase C, X domain containing 2 | PLCXD2 | |
| 12909 | ENSGALG00000016910 | ENSGALT00000027332 | RNA binding motif protein 26 | RBM26 | |
| 13153 | ENSGALG00000016856 | ENSGALT00000030209 | - | - | |

**Table S2.** The total number of adaptively convergent site mutations (ACSMs) in adaptively convergent genes (ACGs) and the number of expected point mutations under neutral selection.

| Locus ID | Locus length (*L*) | Number of ACSMs | Average pairwise p distance (*P*) | Average pairwise Poisson-correction distance (*D*) | Expected number of convergent sites (*M*) |
| --- | --- | --- | --- | --- | --- |
| Nocturnal convergence (H_noc_) | | | | | |
| 175 | 447 | 0 | 0.06984 | 0.07352 | 0.0822 |
| 994 | 786 | 0 | 0.06950 | 0.07250 | 0.1179 |
| 2384 | 1041 | 1 | 0.01992 | 0.02023 | 0.0161 |
| 2812 | 868 | 0 | 0.01382 | 0.01400 | 0.0078 |
| 5023 | 338 | 1 | 0.10637 | 0.11335 | 0.1180 |
| 6857 | 678 | 0 | 0.16277 | 0.18228 | 0.6614 |
| 7499 | 722 | 0 | 0.07347 | 0.07730 | 0.1383 |
| 8089 | 180 | 1 | 0.07160 | 0.07510 | 0.0315 |
| 8190 | 294 | 0 | 0.09517 | 0.10141 | 0.0917 |
| 8420 | 139 | 0 | 0.08377 | 0.08878 | 0.0348 |
| 8613 | 654 | 0 | 0.01478 | 0.01502 | 0.0077 |
| 8696 | 423 | 0 | 0.02054 | 0.02083 | 0.0061 |
| 8704 | 353 | 1 | 0.04178 | 0.04290 | 0.0198 |
| 8758 | 457 | 0 | 0.01250 | 0.01261 | 0.0024 |
| 8813 | 261 | 1 | 0.00440 | 0.00443 | 0.0004 |
| 9012 | 1270 | 2 | 0.16534 | 0.18235 | 1.0798 |
| 9065 | 593 | 1 | 0.14962 | 0.16568 | 0.4762 |
| 9310 | 391 | 0 | 0.02613 | 0.02666 | 0.0102 |
| 10536 | 728 | 0 | 0.04990 | 0.05140 | 0.0546 |
| 11180 | 307 | 0 | 0.04569 | 0.04691 | 0.0187 |
| 12100 | 99 | 0 | 0.09963 | 0.10777 | 0.0403 |
| 13780 | 133 | 0 | 0.02581 | 0.02632 | 0.0034 |
| 13944 | 427 | 0 | 0.02163 | 0.02199 | 0.0077 |
| 14475 | 459 | 1 | 0.03341 | 0.03414 | 0.0166 |
| Total site number | | 9 | - | - | 3.0440 |
| Foot propelled-diving convergence (H_foot_) | | | | | |
| 722 | 1014 | 0 | 0.02165 | 0.02196 | 0.00000 |
| 1152 | 2499 | 0 | 0.02743 | 0.02790 | 0.00000 |
| 2138 | 282 | 0 | 0.11242 | 0.12003 | 0.00004 |
| 2917 | 628 | 0 | 0.05596 | 0.05782 | 0.00001 |
| 4782 | 435 | 0 | 0.03468 | 0.03549 | 0.00000 |
| 5339 | 302 | 0 | 0.03219 | 0.03295 | 0.00000 |
| 6284 | 93 | 1 | 0.03667 | 0.03794 | 0.00000 |
| 8871 | 532 | 0 | 0.01074 | 0.01082 | 0.00000 |
| 9107 | 511 | 2 | 0.00953 | 0.00966 | 0.00000 |
| 10699 | 537 | 0 | 0.08562 | 0.08990 | 0.00002 |
| 10986 | 344 | 0 | 0.03473 | 0.03562 | 0.00000 |
| 12677 | 150 | 0 | 0.10273 | 0.10951 | 0.00002 |
| 12978 | 205 | 0 | 0.11339 | 0.12171 | 0.00004 |
| Total site number | | 3 | - | - | 0.0001 |
| Diurnal raptorial convergence (H_rap_) | | | | | |
| 908 | 638 | 0 | 0.03626 | 0.03706 | 0.0255 |
| 8493 | 585 | 0 | 0.21650 | 0.25090 | 1.0062 |
| 10216 | 407 | 1 | 0.03869 | 0.03969 | 0.0204 |
| 12265 | 310 | 0 | 0.05860 | 0.06060 | 0.0310 |
| 12909 | 1011 | 0 | 0.01085 | 0.01092 | 0.0035 |
| 13153 | 334 | 0 | 0.00906 | 0.00914 | 0.0012 |
| Total site number | | 1 | - | - | 1.0878 |
|  | |  |  |  |  |

**Table S3.** Sequence-convergent genes that show positive selection signals in parts or all of the foreground branches regarding nocturnal, foot-propelled diving or raptorial life history traits. Locus numbers correspond to the numerical IDs of the aligned gene datasets (48_birds_gene_alignment) of Zhang et al. [33], downloaded from the GigagScience Database (http://gigadb.org/dataset/101000). The ENSEMBL transcript, gene IDs and gene names come from the chick (*Gallus gallus*).

| loci | ENSEMBL_Transcript_ID | ENSEMBL_GENE_ID | Full name | Abbr. name | Foreground branch(es) under positive selection |
| --- | --- | --- | --- | --- | --- |
| H_noc_ | | | | | |
| 175 | ENSGALT00000000849 | ENSGALG00000000607 | G protein-coupled receptor 37 like 1 | GPR37L1 | TYTAL and CAPCA |
| 987 | ENSGALT00000004893 | ENSGALG00000003096 | core-binding factor, runt domain, alpha subunit 2; translocated to, 2 | CBFA2T2 | TYTAL |
| 994 | ENSGALT00000004933 | ENSGALG00000003120 | nuclear receptor corepressor 2 | NCOR2 | TYTAL and CAPCA; TYTAL |
| 1158 | ENSGALT00000005705 | ENSGALG00000003604 | solute carrier family 46 member 1 | SLC46A1 | TYTAL |
| 1383 | ENSGALT00000006951 | ENSGALG00000004358 | archaelysin family metallopeptidase 1 | AMZ1 | TYTAL |
| 2384 | ENSGALT00000012177 | ENSGALG00000007528 | transcription elongation regulator 1 | TCERG1 | TYTAL and CAPCA; CAPCA |
| 2465 | ENSGALT00000012644 | ENSGALG00000007796 | breast carcinoma amplified sequence 1 | BCAS1 | TYTAL |
| 2812 | ENSGALT00000014471 | ENSGALG00000008898 | glutamate receptor, ionotropic, N-methyl D-aspartate 1 | GRIN1 | TYTAL and CAPCA |
| 2901 | ENSGALT00000014982 | ENSGALG00000009201 | cyclin F | CCNF | CAPCA |
| 3045 | ENSGALT00000015809 | ENSGALG00000009712 | Uncharacterized protein; Gallus gallus family with sequence similarity 175, member B (FAM175B), mRNA | FAM175B | CAPCA |
| 3138 | ENSGALT00000016420 | ENSGALG00000010096 | MAP3K12 binding inhibitory protein 1 | MBIP | CAPCA |
| 5023 | ENSGALT00000027621 | ENSGALG00000017098 | general transcription factor IIIA | GTF3A | TYTAL and CAPCA |
| 6857 | ENSGALT00000040435 | ENSGALG00000001085 | adhesion G protein-coupled receptor G1 | ADGRG1 | TYTAL and CAPCA; TYTAL |
| 7499 | ENSGALT00000002308 | ENSGALG00000001518 | zinc finger protein 750 | ZNF750 | TYTAL and CAPCA; CAPCA |
| 7664 | ENSGALT00000003144 | ENSGALG00000002020 | glutamate dehydrogenase 1 | GLUD1 | TYTAL |
| 8089 | ENSGALT00000005307 | ENSGALG00000003355 | NOP16 nucleolar protein | NOP16 | TYTAL and CAPCA |
| 8190 | ENSGALT00000005786 | ENSGALG00000003651 | 5'-nucleotidase, cytosolic IIIB | NT5C3B | TYTAL and CAPCA; TYTAL |
| 8420 | ENSGALT00000006803 | ENSGALG00000004279 | galectin-related inter-fiber protein | GRIFIN | TYTAL and CAPCA; TYTAL |
| 8589 | ENSGALT00000007539 | ENSGALG00000004734 | protein eva-1 homolog C-like | LOC422179 | TYTAL |
| 8613 | ENSGALT00000007652 | ENSGALG00000004795 | - | - | TYTAL and CAPCA; TYTAL |
| 8696 | ENSGALT00000008126 | ENSGALG00000005070 | adaptor-related protein complex 3, mu 1 subunit | AP3M1 | TYTAL and CAPCA |
| 8704 | ENSGALT00000008165 | ENSGALG00000005092 | histone deacetylase 11 | HDAC11 | TYTAL and CAPCA |
| 8758 | ENSGALT00000008419 | ENSGALG00000005241 | dynein, cytoplasmic 1, light intermediate chain 2 | DYNC1LI2 | TYTAL and CAPCA; TYTAL |
| 8813 | ENSGALT00000008678 | ENSGALG00000005403 | dehydrogenase/reductase (SDR family) member 11 | DHRS11 | TYTAL and CAPCA |
| 9012 | ENSGALT00000009581 | ENSGALG00000005958 | tripartite motif containing 66 | TRIM66 | TYTAL and CAPCA |
| 9065 | ENSGALT00000009815 | ENSGALG00000006083 | lamin-L(III)-like | LMINA | TYTAL and CAPCA; TYTAL |
| 9310 | ENSGALT00000010951 | ENSGALG00000006769 | Kin17 DNA and RNA binding protein | KIN | TYTAL and CAPCA |
| 9600 | ENSGALT00000012438 | ENSGALG00000007672 | protein tyrosine phosphatase, receptor type, f polypeptide, interacting protein (liprin), alpha 1 | PPFIA1 | TYTAL |
| 10048 | ENSGALT00000014512 | ENSGALG00000008928 | uncharacterized LOC421285 | LOC421285 | CAPCA |
| 10265 | ENSGALT00000015441 | ENSGALG00000009483 | MAP/microtubule affinity-regulating kinase 1 | MARK1 | CAPCA |
| 10536 | ENSGALT00000016557 | ENSGALG00000010186 | calpain 11 | CAPN11 | TYTAL and CAPCA; TYTAL |
| 11180 | ENSGALT00000019496 | ENSGALG00000011952 | solute carrier family 25 member 38 | SLC25A38 | TYTAL and CAPCA |
| 11944 | ENSGALT00000023416 | ENSGALG00000014505 | fibroblast growth factor binding protein 1 | FGFBP1 | TYTAL |
| 12021 | ENSGALT00000023834 | ENSGALG00000014772 | Tryptophanyl tRNA synthetase 2, mitochondrial; Belongs to the class-I aminoacyl-tRNA synthetase family | WARS2 | CAPCA |
| 12100 | ENSGALT00000024177 | ENSGALG00000014989 | S100 calcium binding protein Z | S100Z | TYTAL and CAPCA |
| 13780 | ENSGALT00000036436 | ENSGALG00000017152 | - | - | TYTAL and CAPCA; CAPCA |
| 13798 | ENSGALT00000036563 | ENSGALG00000016822 | Sodium/potassium-transporting ATPase subunit beta | ATP4B | TYTAL |
| 13910 | ENSGALT00000037252 | ENSGALG00000022976 | Orofacial cleft 1 candidate gene 1 protein homolog | OFCC1 | CAPCA |
| 13944 | ENSGALT00000037439 | ENSGALG00000012327 | inhibin, beta A | INHBA | TYTAL and CAPCA |
| 14475 | ENSGALT00000040340 | ENSGALG00000007889 | Coiled-coil domain containing 85A | CCDC85A | TYTAL and CAPCA |
| H_foot_ | | |  |  |  |
| 710 | ENSGALT00000003463 | ENSGALG00000002212 | RAD51 paralog D | RAD51D | PHACA |
| 722 | ENSGALT00000003553 | ENSGALG00000002266 | oxoglutarate dehydrogenase-like | OGDHL | PHACA, GAVST and PODCR; PODCR |
| 1152 | ENSGALT00000005664 | ENSGALG00000003578 | fibronectin 1 | FN1 | PHACA, GAVST and PODCR; PHACA; GAVST |
| 2138 | ENSGALT00000010970 | ENSGALG00000006782 | mitochondrial ribosomal protein L46 | MRPL46 | PHACA, GAVST and PODCR; GAVST |
| 2917 | ENSGALT00000015069 | ENSGALG00000009258 | KIAA1598-like | LOC423919 | PHACA, GAVST and PODCR |
| 3064 | ENSGALT00000015952 | ENSGALG00000009807 | feline leukemia virus subgroup C cellular receptor 1 | FLVCR1 | PHACA |
| 3314 | ENSGALT00000017381 | ENSGALG00000010684 | - | - | PHACA, GAVST |
| 4782 | ENSGALT00000026329 | ENSGALG00000016320 | F-box protein 9 | FBXO9 | PHACA, GAVST and PODCR |
| 4903 | ENSGALT00000005933 | ENSGALG00000016731 | mitochondrial ribosomal protein L19 | MRPL19 | GAVST |
| 5339 | ENSGALT00000031453 | ENSGALG00000000302 | troponin T type 2 (cardiac) | TNNT2 | PHACA, GAVST and PODCR; PHACA; PODCR |
| 5597 | ENSGALT00000010037 | ENSGALG00000005411 | meiosis specific with OB domains | MEIOB | GAVST |
| 6284 | ENSGALT00000038066 | ENSGALG00000011566 | glycophorin C (Gerbich blood group) | GYPC | PHACA, GAVST and PODCR |
| 7660 | ENSGALT00000003133 | ENSGALG00000002012 | multimerin 2 | MMRN2 | PODCR |
| 8170 | ENSGALT00000005672 | ENSGALG00000003584 | karyopherin alpha 2 (RAG cohort 1, importin alpha 1) | KPNA2 | PHACA |
| 8219 | ENSGALT00000005933 | ENSGALG00000003739 | sal-like 1 (Drosophila) | SALL1 | PODCR |
| 8249 | ENSGALT00000013045 | ENSGALG00000003824 | calcium homeostasis endoplasmic reticulum protein | CHERP | GAVST |
| 8871 | ENSGALT00000008906 | ENSGALG00000005552 | polypyrimidine tract binding protein 2 | PTBP2 | PHACA, GAVST and PODCR |
| 9107 | ENSGALT00000010037 | ENSGALG00000006220 | potassium voltage-gated channel, Shaw-related subfamily, member 1 | KCNC1 | PHACA, GAVST and PODCR; PHACA; PODCR |
| 9654 | ENSGALT00000025302 | ENSGALG00000007818 | - | - | GAVST |
| 9726 | ENSGALT00000013045 | ENSGALG00000008039 | major facilitator superfamily domain containing 13A | MFSD13A | PODCR |
| 10699 | ENSGALT00000017326 | ENSGALG00000010652 | sterol carrier protein 2 | SCP2 | PHACA, GAVST and PODCR; PHACA |
| 10986 | ENSGALT00000018629 | ENSGALG00000011419 | Dual specificity protein phosphatase 4 | DUSP4 | PHACA, GAVST and PODCR |
| 11092 | ENSGALT00000019120 | ENSGALG00000011697 | Gallus gallus SEC22 vesicle trafficking protein homolog A | SEC22A | PHACA |
| 11371 | ENSGALT00000020433 | ENSGALG00000012508 | thyroid hormone receptor interactor 13 | TRIP13 | PHACA |
| 11559 | ENSGALT00000021346 | ENSGALG00000013072 | aldo-keto reductase family 1, member B10 (aldose reductase) | AKR1B10 | GAVST |
| 12407 | ENSGALT00000025302 | ENSGALG00000015688 | MAX dimerization protein 4 | MXD4 | PODCR |
| 12677 | ENSGALT00000026366 | ENSGALG00000016347 | apolipoprotein O | APOO | PHACA, GAVST and PODCR |
| 12799 | ENSGALT00000026845 | ENSGALG00000016630 | exostosin-like glycosyltransferase 3 | EXTL3 | PHACA |
| 12978 | ENSGALT00000027620 | ENSGALG00000017097 | mitochondrial translational initiation factor 3 | MTIF3 | PHACA, GAVST and PODCR |
| H_rap_ | |  |  |  |  |
| 908 | ENSGALT00000004538 | ENSGALG00000002878 | CREB3 regulatory factor | CREBRF | CATAU, HALLE, HALAL and FALPE; CATAU, HALLE and HALAL |
| 3984 | ENSGALT00000021382 | ENSGALG00000013097 | solute carrier family 15, member 5(SLC15A5) | SLC15A5 | CATAU, HALLE and HALAL |
| 8221 | ENSGALT00000005954 | ENSGALG00000003750 | phospholipase C, gamma 1(PLCG1) | PLCG1 | CATAU, HALLE and HALAL |
| 8493 | ENSGALT00000007125 | ENSGALG00000004475 | - | - | CATAU, HALLE, HALAL and FALPE; FALPE |
| 9996 | ENSGALT00000014246 | ENSGALG00000008757 | cannabinoid receptor interacting protein 1 | CNRIP1 | FALPE |
| 10216 | ENSGALT00000015264 | ENSGALG00000009370 | - | - | CATAU, HALLE, HALAL and FALPE |
| 10719 | ENSGALT00000017396 | ENSGALG00000010697 | Semaphorin-4D | SEMA4D | FALPE |
| 11588 | ENSGALT00000021512 | ENSGALG00000013171 | coiled-coil domain containing 127 | CCDC127 | FALPE |
| 12265 | ENSGALT00000024806 | ENSGALG00000015375 | phosphatidylinositol-specific phospholipase C, X domain containing 2 | PLCXD2 | CATAU, HALLE, HALAL and FALPE |
| 12909 | ENSGALT00000027332 | ENSGALG00000016910 | RNA binding motif protein 26 | RBM26 | CATAU, HALLE, HALAL and FALPE; CATAU, HALLE and HALAL |
| 13153 | ENSGALT00000030209 | ENSGALG00000016856 | - | - | CATAU, HALLE, HALAL and FALPE; CATAU, HALLE and HALAL |

**Table S4.** Adaptively convergent genes supporting for one of the derived convergence hypotheses (H_foot-a_, H_foot-b_, H_foot-c_, H_rap-a_ and H_rap-b_, see Fig. 3). Locus numbers correspond to the numerical IDs of the aligned gene datasets (48_birds_gene_alignment) of Zhang et al. [33], downloaded from the GigagScience Database (http://gigadb.org/dataset/101000). The ENSEMBL transcript, gene IDs and gene names come from the chick (*Gallus gallus*).

| Locus | ENSEMBL_Transcript_ID | ENSEMBL_GENE_ID | Full name | Abbr. name |
| --- | --- | --- | --- | --- |
| H_foot-a_ (loon-grebe) | | | | |
| 482 | ENSGALT00000002261 | ENSGALG00000001491 | non-SMC condensin II complex subunit D3 | NCAPD3 |
| 1338 | ENSGALT00000006717 | ENSGALG00000021039 | hexokinase domain containing 1 | HKDC1 |
| 1471 | ENSGALT00000007401 | ENSGALG00000004646 | F-box protein 6 | FBXO6 |
| 5339 | ENSGALT00000031453 | ENSGALG00000000302 | troponin T type 2 (cardiac) | TNNT2 |
| 8696 | ENSGALT00000008126 | ENSGALG00000005070 | adaptor-related protein complex 3, mu 1 subunit | AP3M1 |
| 9107 | ENSGALT00000010037 | ENSGALG00000006220 | potassium voltage-gated channel, Shaw-related subfamily, member 1 | KCNC1 |
| 9267 | ENSGALT00000010756 | ENSGALG00000006649 | transmembrane protein 41A | TMEM41A |
| 10448 | ENSGALT00000016184 | ENSGALG00000009957 | tyrosine kinase with immunoglobulin-like and EGF-like domains 1 | TIE1 |
| 11000 | ENSGALT00000018679 | ENSGALG00000011456 | cyclin-dependent kinase 17 | CDK17 |
| 12391 | ENSGALT00000025248 | ENSGALG00000015661 | cysteine-rich secretory protein LCCL domain containing 1 | CRISPLD1 |
| 12791 | ENSGALT00000026803 | ENSGALG00000016607 | chloride channel, voltage-sensitive 4 | CLCN4 |
| 13612 | ENSGALT00000034634 | ENSGALG00000003221 | WD repeat domain 61 | WDR61 |
| H_foot-b_ (cormorant-grebe) | | | | |
| 183 | ENSGALT00000000894 | ENSGALG00000000628 | Kinesin-like protein; Kinesin family member 21B | KIF21B |
| 1146 | ENSGALT00000005653 | ENSGALG00000003570 | 5-aminoimidazole-4-carboxamide ribonucleotide formyltransferase/IMP cyclohydrolase | ATIC |
| 1471 | ENSGALT00000007401 | ENSGALG00000004646 | F-box protein 6 | FBXO6 |
| 2544 | ENSGALT00000012966 | ENSGALG00000007988 | alkB homolog 3, alpha-ketoglutarate-dependent dioxygenase | ALKBH3 |
| 4111 | ENSGALT00000022268 | ENSGALG00000019514 | CNDP dipeptidase 2 (metallopeptidase M20 family) | CNDP2 |
| 5339 | ENSGALT00000031453 | ENSGALG00000000302 | troponin T type 2 (cardiac) | TNNT2 |
| 7631 | ENSGALT00000003000 | ENSGALG00000001938 | solute carrier family 25 (mitochondrial carrier; phosphate carrier), member 24 | SLC25A24 |
| 8417 | ENSGALT00000006794 | ENSGALG00000004273 | Ral GEF with PH domain and SH3 binding motif 2 | RALGPS2 |
| 9107 | ENSGALT00000010037 | ENSGALG00000006220 | potassium voltage-gated channel, Shaw-related subfamily, member 1 | KCNC1 |
| 12673 | ENSGALT00000026343 | ENSGALG00000016331 | squalene epoxidase | SQLE |
| 13005 | ENSGALT00000027725 | ENSGALG00000017165 | cullin 5 | CUL5 |
| 13198 | ENSGALT00000030570 | ENSGALG00000012698 | - | - |
| H_foot-c_ (loon-cormorant) | | | | |
| 710 | ENSGALT00000003463 | ENSGALG00000002212 | RAD51 paralog D | RAD51D |
| 2025 | ENSGALT00000010317 | ENSGALG00000006388 | interleukin 16 | IL16 |
| 2138 | ENSGALT00000010970 | ENSGALG00000006782 | mitochondrial ribosomal protein L46 | MRPL46 |
| 2857 | ENSGALT00000014668 | ENSGALG00000009016 | SLX4 interacting protein | SLX4IP |
| 2917 | ENSGALT00000015069 | ENSGALG00000009258 | KIAA1598-like | LOC423919 |
| 7468 | ENSGALT00000002174 | ENSGALG00000001426 | mitochondrial trans-2-enoyl-CoA reductase | MECR |
| 9107 | ENSGALT00000010037 | ENSGALG00000006220 | potassium voltage-gated channel, Shaw-related subfamily, member 1 | KCNC1 |
| 10631 | ENSGALT00000017025 | ENSGALG00000010456 | kelch repeat-containing protein 2-like | LOC772017 |
| 10699 | ENSGALT00000017326 | ENSGALG00000010652 | sterol carrier protein 2 | SCP2 |
| 12232 | ENSGALT00000024674 | ENSGALG00000015287 | leukemia NUP98 fusion partner 1 | LNP1 |
| H_rap-a_ (falcon-vulture) | | | | |
| 171 | ENSGALT00000000839 | ENSGALG00000000600 | protein tyrosine phosphatase, non-receptor type 7 | PTPN7 |
| 1207 | ENSGALT00000005972 | ENSGALG00000003763 | interactor of little elongation complex ELL subunit 2 | ICE2 |
| 2241 | ENSGALT00000011479 | ENSGALG00000007087 | - | - |
| 2592 | ENSGALT00000013198 | ENSGALG00000008132 | pseudouridylate synthase 7 (putative) | PUS7 |
| 2798 | ENSGALT00000014395 | ENSGALG00000008862 | DnaJ heat shock protein family (Hsp40) member C10 | DNAJC10 |
| 3696 | ENSGALT00000019695 | ENSGALG00000012057 | tetratricopeptide repeat and ankyrin repeat containing 1 | TRANK1 |
| 3882 | ENSGALT00000020725 | ENSGALG00000012696 | RAN binding protein 9 | RANBP9 |
| 4971 | ENSGALT00000027385 | ENSGALG00000016943 | olfactomedin 4 | OLFM4 |
| 5103 | ENSGALT00000028047 | ENSGALG00000017363 | chromosome 11 open reading frame, human C16orf87 | C11H16ORF87 |
| 5149 | ENSGALT00000029195 | ENSGALG00000011635 | transcription factor CP2-like 1 | TFCP2L1 |
| 7723 | ENSGALT00000003408 | ENSGALG00000002175 | kinesin family member 14 | KIF14 |
| 9157 | ENSGALT00000010272 | ENSGALG00000006355 | SPT2 chromatin protein domain containing 1 | SPTY2D1 |
| 12942 | ENSGALT00000027473 | ENSGALG00000017008 | regulator of chromosome condensation (RCC1) and BTB (POZ) domain containing protein 1 | RCBTB1 |
| 13580 | ENSGALT00000034267 | ENSGALG00000021260 | - | - |
| 13611 | ENSGALT00000034628 | ENSGALG00000021452 | tetraspanin-3-like | LOC101748661 |
| 13989 | ENSGALT00000037720 | ENSGALG00000011171 | TATA box binding protein | TBP |
| H_rap-b_ (falcon-eagle) | | | | |
| 2668 | ENSGALT00000013671 | ENSGALG00000008392 | cilia and flagella associated protein 43 | CFAP43 |
| 3496 | ENSGALT00000018508 | ENSGALG00000011342 | FYVE, RhoGEF and PH domain containing 6 | FGD6 |
| 6475 | ENSGALT00000038876 | ENSGALG00000001565 | Complement component 5 | C5 |
| 8451 | ENSGALT00000006970 | ENSGALG00000004369 | pygopus family PHD finger 1 | PYGO1 |
| 9213 | ENSGALT00000010504 | ENSGALG00000006501 | gamma-glutamyltransferase 5 | GGT5 |
| 9473 | ENSGALT00000011753 | ENSGALG00000007261 | - | - |
| 10975 | ENSGALT00000018551 | ENSGALG00000011372 | cyclin-dependent kinase 2 interacting protein | CINP |
| 11301 | ENSGALT00000020078 | ENSGALG00000012285 | BAI1 associated protein 2 like 2 | BAIAP2L2 |
| 12627 | ENSGALT00000026140 | ENSGALG00000016216 | N-myc downstream regulated 1 | NDRG1 |
| 13413 | ENSGALT00000032636 | ENSGALG00000009050 | calpain 3, (p94) | CAPN3 |
| 13611 | ENSGALT00000034628 | ENSGALG00000021452 | tetraspanin-3-like | LOC101748661 |
| 14179 | ENSGALT00000038745 | ENSGALG00000009305 | Lamin-B receptor | LBR |

**Figure S1.** Maximum likelihood gene trees of 43 adaptively convergent genes, supporting one of the tree convergent hypotheses – nocturnality (H_noc_), foot-propelled diving (H_foot_) and raptorial behaviors (H_rap_). The focal taxa with each convergent trait are marked as red color. Gene trees with the focal convergent taxa forming a monophyly are shaded in grey.
